# Prevalence and genetic characterization of *Cryptosporidium*, *Enterocytozoon*, *Giardia* and *Cyclospora* in diarrheal outpatients in china

**DOI:** 10.1186/1471-2334-14-25

**Published:** 2014-01-13

**Authors:** Hua Liu, Yujuan Shen, Jianhai Yin, Zhongying Yuan, Yanyan Jiang, Yuxin Xu, Wei Pan, Yuan Hu, Jianping Cao

**Affiliations:** 1 National Institute of Parasitic Diseases, Chinese Center for Disease Control and Prevention, Shanghai, People’s Republic of China; 2 Key Laboratory of Parasite and Vector Biology, Ministry of Health, Shanghai, People’s Republic of China; 3 WHO Collaborating Center for Malaria, Schistosomiasis and Filariasis, Shanghai, People’s Republic of China

**Keywords:** *Cryptosporidium*, *Enterocytozoon*, *Giardia*, *Cyclospora*, Outpatients, Genotype

## Abstract

**Background:**

*Cryptosporidium* spp., *Enterocytozoon* spp., *Giardia* spp. and *Cyclospora* spp. are important intestinal protozoan parasites causing diarrhea in humans, livestocks and wildlife and have a significant impact on public health. No reports exist about simultaneous prevalence rates or genotyping data of these four parasites in outpatients from China.

**Methods:**

Fecal specimens from 252 diarrhea patients in a pediatric clinic (n = 169) and an intestinal clinic (n = 83) of a hospital in Shanghai, China, were collected between October 2012 and March 2013. All samples were examined for the presence of the four parasites by using molecular methods.

**Results:**

In total, 76/252 (30.16%) patients were positive for at least one intestinal parasite, of which *Cryptosporidium* spp., *Enterocytozoon bieneusi* and *Giardia intestinalis* were detected by nested PCR in 34 (13.49%), 34 (13.49%) and 17 (6.75%) of the fecal specimens, respectively. Sequence analysis showed that all *Cryptosporidium-*positive specimens were *C. andersoni* and that most *G. intestinalis*- positive patients were infected by assemblage C, which is usually found in canids, while only one sample was from assemblage B. Eight patients were co-infected with *Cryptosporidium* spp. and *Enterocytozoon*, while one was co-infected with *Cryptosporidium* and *Giardia*.

**Conclusions:**

The patients infected with *Cryptosporidium* and *Enterocytozoon bieneusi* had higher infection rates in winter than in spring in this area. Data indicated that *C. andersoni* is the fourth major *Cryptosporidium* species infecting humans in addition to *C. hominis*, *C. parvum* and *C. meleagridis*. Our study also revealed a short-term outbreak of cryptosporidiosis and microsporidiosis and sporadic cases of giardiasis that occurred among humans in Shanghai, China.

## Background

Cryptosporidiosis, microsporidiosis, giardiasis and cyclosporiasis are emerging infectious diseases. *Cryptosporidium* spp., *Enterocytozoon* spp., *Giardia* spp. and *Cyclospora* spp. are diarrhea-causing intestinal protozoans of humans (especially in AIDS patients), livestock and wildlife worldwide [1-4]. Although they are considered opportunistic pathogens, they have caused several outbreaks and emergency responses among humans and animals [5-9] These four parasites pose significant challenges to public health and water authorities, especially in developing countries because of the high prevalence and disease burden of the infections [10,11]. Furthermore, they exert negative pressure on the growth and cognitive functions of infected children and immunocompromised persons [1,12,13].

*Cryptosporidium* and microsporidia infect AIDS or immunodeficient patients, travelers, children and the elderly. *C. parvum* and *C. hominis* are the main human-pathogenic *Cryptosporidium* species, while *C. muris* and *C. andersoni* are minor zoonotic species, for which small numbers of human cases have recently been reported [14,15]. The prevalent microsporidia species *E. bieneusi* has been most frequently identified in human clinical fecal samples as well as in wild and domestic animals [16,17]. Molecular diagnostic tools have been used to trace the source of human infections and transmissions, thus confirming its zoonotic potential [18].

*G. intestinalis* is the etiologic agent of giardiasis, a common gastrointestinal disease in humans, livestock and companion animals. *G. intestinalis* is considered as a complex species and based on genetic analysis has been grouped into eight assemblages (A–H) [19,20]. Both assemblages A and B, which can be transmitted zoonotically, have a wide host range and are responsible for human infections [21,22]. Assemblages C–G appear to be strictly host-specific: C and D are found largely in canids, E in domestic mammals, F in cats, G in rodents and H in seals [1,23].

*Cyclospora cayetanensis*, an emerging human pathogen, causes severe diarrheal disease and has resulted in several foodborne outbreaks in humans [2,24,25]. The transmission dynamics of this parasite are still unknown. In previous studies, feces as well as contaminated water sources were considered as transmission routes [26,27].

Currently, no reports exist about simultaneous prevalence rates and genotyping data of cryptosporidiosis, microsporidiosis, giardiasis and cyclosporiasis in China. Therefore, this study focused on the prevalence and genetic characterizations of the four diseases in diarrhea outpatients of a pediatric clinic and an intestinal clinic in Shanghai and assessed their potential zoonotic transmission.

## Methods

### Ethical statement

Ethical clearance for the collection and examination of human feces samples was obtained from the Ethics Committee of the National Institute of Parasitic Diseases, Chinese Center for Disease Control and Prevention, China (reference no. 2012–12). The objectives, procedures and potential risk were orally explained to all participants. Written informed consent was given to, and signed by all participating in the study. Parents/guardians provided consent on behalf of all infant participants.

### Specimen collection and DNA extraction

Fecal specimens from 252 diarrhea patients in a pediatric clinic (n = 169) and an intestinal clinic (n = 83) of a hospital in Pudong, Shanghai, China, were collected between October 2012 and March 2013. Patient details, including their age, gender, address, frequency of diarrhea and consistency of stools, were recorded. Specimens were collected from patients with fecal excretion heavier than 200 g and with no less than three events of diarrhea per day. The stools consistency was usually thin and mixed with mucus or blood. Sufficient samples were collected for DNA extraction and purification using the QIAamp DNA stool Mini Kit (QIAGEN, Hilden, Germany). The extracted DNA was stored at -30°C for polymerase chain reaction (PCR).

### Parasite identification in clinical samples

The small subunit (SSU) rRNA gene of *Cryptosporidium* was identified using a nested PCR [28]. The presence of *E. bieneusi*, *G. intestinalis* and *Cyclospora* in the specimens was detected using individual nested PCR and sequence analysis of the SSU rRNA gene [29], the triose phosphate isomerase (TPI) gene [30] and the 18S rRNA gene [31], respectively.

All primers used in the study are listed in Table 1. Go Taq**®** Green Master Mix (containing Go Taq® DNA Polymerase, dNTP mixture, Green Go Taq Reaction Buffer, MgCl_2_; Promega) was used to amplify the genes of *Cryptosporidium* and *G. intestinalis*, while *Premix Taq***®** (containing Taq DNA Polymerase, dNTP mixture, *Taq* Buffer, Tartrazine/Xylene Cyanol FF; Takara) was used to identify *Enterocytozoon* and *Cyclospora* genes. Each 25 μl reaction mixture contained 12.5 μl Taq mix, 1 μl of 10 μM sense and antisense primers each, 1 μl DNA template and 12.5 μl nuclease-free water.

**Table 1:** Primers used for protozoan gene amplification

**Genus**	**Gene**	**Sequence of primers (5´ → 3´)**	**Amplicon size (bp)**
*Cryptosporidium*	SSUrRNA	CryF1: TTCTAGAGCTAATACATGCG	~1325
		CryR1: CCATTTCCTTCGAAACAGGA	
		CryF2: GGAAGGGTTGTATTTATTAGATAAAG	~840
		CryR2: CTCATAAGGTGCTGAAGGAGTA	
Microsporidia	SSUrRNA	EF1: GATGGTCATAGGGATGAAGAGCTT	~1200
		ER1: AATACAGGATCACTTGGATCCGT	
		EF2: AGGGATGAAGAGCTTCGGCTCTG	~600
		ER2: AATATCCCTAATACAGGATCACT	
*Giardia*	TPI	TPIF1: AAATIATGCCTGCTCGTCG	~605
		TPIR1: CAAACCTTITCCGCAAACC	
		TPIF2: CCCTTCATCGGIGGTAACTT	~530
		TPIR2: GTGGCCACCACICCCGTGCC	
*Cyclospora*	18SrRNA	CYCLF1: AATGTAAAACCCTTCCAGAGTAAC	~1000
		CYCLR1: GCAATA ATCTATCCCCATCACG	
		CYCLF2: AATTCCAGCTCCAATAGTGTAT	~500
		CYCLR2: CAGGAGAAGCCAAGGTAGGCRTTT	

The thermal profile of *Cryptosporidium* PCR consisted of 94°C for 1 min, 35 cycles of 94°C for 50 s, 55°C for 30 s and 72°C for 1 min, followed by 72°C for 10 min, with a hold step at 4°C. A second reaction was carried out similarly. Each specimen was analyzed at least three times by PCR with positive and negative controls in each run. The other amplification conditions varied in annealing temperature and extension time. For *Enterocytozoon*, the annealing step was at 57.4°C, and the extension step was at 72°C for 90 s; the annealing step for *Giardia* was at 57.5°C, and the extension step was at 72°C for 1 min. The cycling conditions for *Cyclospora* were as follows: the primary cycle consisted of 94°C for 1 min, 35 cycles of 94°C for 50 s, 56°C for 30 s and 72°C for 90 s, followed by 72°C for 10 min, and termination at 4°C. The secondary step differed in extension time (72°C for 1 min).

### Sequencing of each gene

Secondary PCR products were directly sequenced on an ABI 3730 DNA Analyzer (Applied Biosystems, Foster City, USA) using the secondary primers and a Big Dye Terminator v3.1 Cycle Sequencing kit (Applied Biosystems). The sequence accuracy was confirmed by two-directional sequencing and by sequencing a new PCR product if necessary.

### Statistical analysis

ContigExpress was used to assemble sequences. Sequences were aligned using the program ClustalX 1.83 (ftp://ftp-igbmc.u-strasbg.fr/pub/ClustalX/). All statistical analyses were performed using SPSS version 17.0 (SPSS Inc., Chicago, IL). The chi-squqre test was used to analyse the data, with *P* < 0.05 considered to indicate significant differences.

### Nucleotide sequence accession numbers

Representative nucleotide sequences were deposited in GenBank under the accession numbers KF271440 to KF271519.

## Results

### Occurrence of *Cryptosporidium*, *Enterocytozoon*, *Giardia* and *Cyclospora*

*Cryptosporidium* spp., *E. bieneusi* and *G. intestinalis* were detected by nested PCR in 34 (13.49%), 34 (13.49%) and 17 (6.75%) of the 252 fecal specimens, respectively (Table 2). *Cyclospora* was not detected. The *Cryptosporidium*-, *E. bieneusi*- and *Giardia*-positive patients were not restricted to a particular clinic. Polyparasitism was observed in nine of the 252 patients, eight of which were co-infected with *Cryptosporidium* and *Enterocytozoon*, while one was co-infected with *Cryptosporidium* and *Giardia*. No age-associated differences in the patients involved (ranging from 1 month to 77 years) was found in our study.

**Table 2:** **Sex ratio of *Cryptosporidium*, *Enterocytozoon*, *Giardia *** **and *Cyclospora***

**Genus**	**Number of positive specimens (%)**			
	**Male**	**Female**	**Total**	**Species/genotype**
*Cryptosporidium*	23 (15.13)	11 (10.00)	34 (13.49)	*C. andersoni*
*Enterocytozoon*	22 (14.47)	12 (12.00)	34 (13.49)	-
*Giardia*	11 (7.24)	6 (6.00)	17 (6.75)	Assemblage C (16) Assemblage B (1)
*Cyclospora*	0	0	0	-
Total patients	152	100	252	

### *Cryptosporidium* species

**Figure 1:**
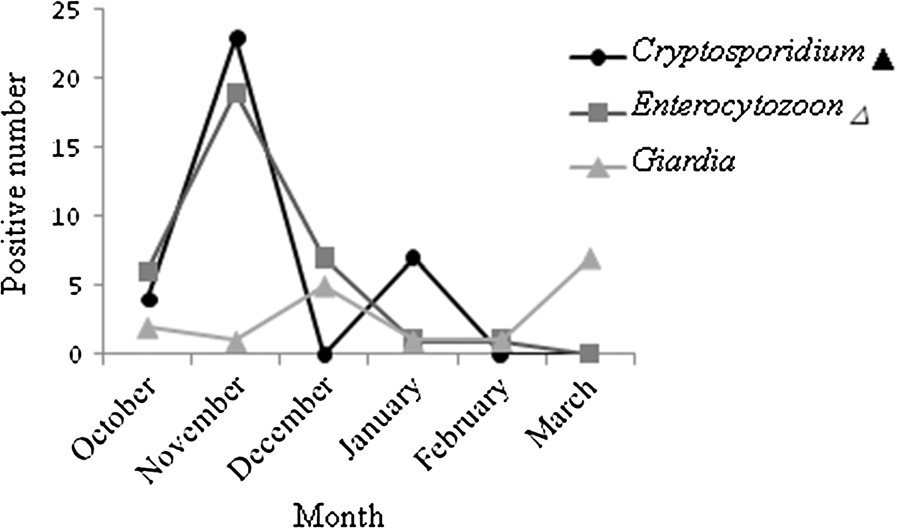
**Seasonal patterns of *Cryptosporidium, Enterocytozoon *and *Giardia *infections in humans.** ▲: Chi-square analysis of seasonal differences in the rates of infection by *Cryptosporidium*: *P* < 0.05 (*P* = 0.024); Δ: Chi-square analysis of seasonal differences in the rates of infection by *Enterocytozoon*: *P* < 0.0001. ▀: Chi-square analysis of seasonal differences in the rates of infection by *Giardia*:*P* > 0.05 (*P* = 0.123).

**Figure 2:**
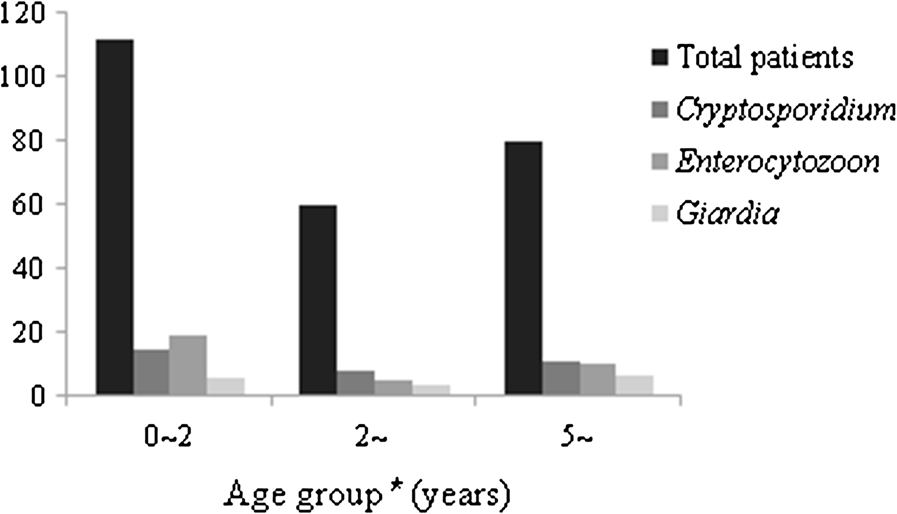
**Age distribution of *Cryptosporidium*-, *Enterocytozoon*- and *Giardia*-positive patients in Shanghai, China.**: total number of patients investigated in different months; : positive cases of *Cryptosporidium*; (dark gray square): *Enterocytozoon*-positive cases; (light gray square): positive cases of *Giardia*; ^*^Chi-square analysis of age differences in the rates of infection by the three parasites: *P* > 0.05.

Sequence analysis of *Cryptosporidium* indicated that all positive specimens belonged to *C. andersoni*, which is usually found in cattle. Patients tested in winter had a higher positivity rate (17.31%) than those tested in spring (7.29%, *P* = 0.024, Figure 1). However, no sex- or age-associated differences in detection rates were found (*P* > 0.05; Figure 2).

### *Enterocytozoon* infections

Based on sequence analysis of the SSU rRNA gene, the microsporidia-positive outpatients were identified as *E. bieneusi*. Univariate analysis did not show any significant age- or sex-associated differences in *E. bieneusi* infection rates. However, patients showed higher detection rates for microsporidiosis in winter than in spring (*P* < 0.05; Figure 1).

### *Giardia* genotyping

Of the 17 *Giardia*-positive patients, most isolates belonged to assemblage C, whereas only one belonged to assemblage B; this result differs from previous reports [4,21]. In addition, we found no differences associated with age, sex or season.

## Discussion

Intestinal parasitic infections remain an important pathogenic factor of diarrhea in developing countries, especially among HIV-positive patients [32]. This study reports the epidemiological and genetic characterizations of four opportunistic intestinal parasites of outpatients from the pediatric clinic and intestinal clinic of a hospital in Shanghai, China. Using nested PCR and sequence analysis in this cross-sectional study, *Cryptosporidium* (13.49%), *Enterocytozoon* (13.49%) and *Giardia* (6.75%) were possible etiologic agents in this area. However, *Cyclospora* was not detected in these patients. A high proportion (3.17%) of polyparasitism was observed. The presence of co-infections was supported by the finding that these patients exhibited the most severe symptoms, with diarrhea frequencies reaching eight episodes per day.

*C. andersoni* is primarily found in cattle or in contaminated water [33,34], while few studies have reported *C. andersoni* infection in humans [15,35]. However, all *Cryptosporidium*-positive patients in this study were found to be infected with *C. andersoni*. The reasons for the occurrence of *C. andersoni* infection might be attributed to the environment, patient populations and geographic locations. Interestingly, our results were consistent with a recent study, which showed *C. andersoni* to be the dominant species in source and tap water in Shanghai [33]. Through genotyping, the researchers concluded that the contamination of source and tap water originated mainly from animal farms, especially, cattle farms. Similarly, *C. andersoni* was the most common species in the patients from our study, and sequence analysis suggested that contaminated water or infected cattles might be the possible transmission routes. In addition, the Yangtze River and Pudong Canal, which run through this area, are used for irrigation, livestock feeding and drinking water for residents. Extensive studies have reported seasonal differences in the distribution of *C. parvum* and *C. hominis* in the United Kingdom and New England, USA [36-38]. In Shanghai, increased *C. andersoni* detection rates during winter may have been due to changes in animal breeding, rainfall, travel and recreational activities.

Although *E. bieneusi* is nowadays considered to be an opportunistic pathogen in HIV-infected or organ transplant recipients, *E. bieneusi* infections have also been found in HIV-negative patients, immunocompetent and other healthy persons [39-42]. A recent study described a healthy man infected with a novel species of *Microsporidium*[43]. In the present study, 11 diarrheal adults (13.25%) and 23 children (13.61%) were detected with *E. bieneusi*, which suggested that this infection was not correlated with age in the study area(*P* > 0.05). However, humans were more susceptible to microsporidiosis in winter than in spring. The reason for this might be a reduced immunity and resistance during winter. Studies have reported *E. bieneusi* to be associated with acute and chronic diarrhea [44,45]. The sources of microsporidia infecting humans and its modes of transmission remain unclear. Due to the release of spores into the environment via stool and respiratory secretions, possible sources of infection might be humans or animals infected with microsporidia [4].

Genotyping results of *G. intestinalis* indicated that all but one positive specimens belonged to assemblage C, which is usually found in canids [46], thus suggesting that dogs may serve as sources of zoonotic transmission of giardiasis in this region. Sequence analyses of the TPI gene revealed that the only specimen from assemblage B had a high homology with an isolate from a primate in Japan [47].

Overall, patients in Shanghai were found to be detected with *C. andersoni*, *E. bieneusi* and *G. intestinalis*. These three intestinal protozoa can be transmitted through the fecal-oral or oral-oral routes, inhalation of aerosols or ingestion of food or water contaminated with fecal material [4,48]. Therefore, it can be speculated that family members may also be infected, although more questionnaires and comprehensive epidemiological investigations are required to confirm this hypothesis. It has been reported that contamination of food or water by animals such as cattle or canids are causes of several foodborne and waterborne outbreaks of cryptosporidiosis [49-51]. In order to better understand the source of infection, we will attempt to seek the cooperation of patients involved in future studies to investigate their habits, such as contact with animals, drinking water and water conditions. In addition, we will continue to monitor the patients from the intestinal clinic and the pediatric clinic to determine whether the observed prevalence rates will persist. We also aim to extend the investigation to family members to determine the existence of a household cluster outbreak.

## Conclusions

Based on our study, intestinal parasites were common among the study population of diarrheal outpatients in this area in Shanghai, China, between October 2012 and March 2013, *C. andersoni*, *E. bieneusi* and *G. intestinalis* (mainly assemblage C) were the major parasite species. The source of these infections remains to be tracked to determine their potential zoonotic transmission route.

## Competing interests

The authors declare that they have no competing interests.

## Authors’ contributions

Conceived and designed the experiments: Y.S. J.C. H.L. Performed the experiments: HL YS JY ZY YX YJ WP YH Analyzed the data: YS HL JY JC Contributed reagents/materials/analysis tools: J.C. Y.S. Wrote the paper: HL YS JC. All authors read and approved the final version of the manuscript.

## Pre-publication history

The pre-publication history for this paper can be accessed here:

http://www.biomedcentral.com/1471-2334/14/25/prepub
